# Constitutively Active 5-HT Receptors: An Explanation of How 5-HT Antagonists Inhibit Gut Motility in Species Where 5-HT is Not an Enteric Neurotransmitter?

**DOI:** 10.3389/fncel.2015.00487

**Published:** 2015-12-18

**Authors:** Nick J. Spencer

**Affiliations:** Department of Human Physiology and Centre for Neuroscience, Flinders University of South AustraliaAdelaide, SA, Australia

**Keywords:** peristalsis, enteric nervous system, migrating motor complex, colon, serotonin, 5-HT, colonic transit

## Abstract

Antagonists of 5-Hydroxytryptamine (5-HT) receptors are well known to inhibit gastrointestinal (GI)-motility and transit in a variety of mammals, including humans. Originally, these observations had been interpreted by many investigators (including us) as evidence that endogenous 5-HT plays a major role in GI motility. This seemed a logical assumption. However, the story changed dramatically after recent studies revealed that 5-HT antagonists still blocked major GI motility patterns (peristalsis and colonic migrating motor complexes) in segments of intestine depleted of all 5-HT. Then, these results were further supported by Dr. Gershons' laboratory, which showed that genetic deletion of all genes that synthesizes 5-HT had minor, or no inhibitory effects on GI transit *in vivo*. If 5-HT was essential for GI motility patterns and transit, then one would expect major disruptions in motility and transit when 5-HT synthesis was genetically ablated. This does not occur. The inhibitory effects of 5-HT antagonists on GI motility clearly occur independently of any 5-HT in the gut. Evidence now suggests that 5-HT antagonists act on 5-HT receptors in the gut which are constitutively active, and don't require 5-HT for their activation. This would explain a long-standing mystery of how 5-HT antagonists inhibit gut motility in species like mice, rats, and humans where 5-HT is not an enteric neurotransmitter. Studies are now increasingly demonstrating that the presence of a neurochemical in enteric neurons does not mean they function as neurotransmitters. Caution should be exercised when interpreting any inhibitory effects of 5-HT antagonists on GI motility.

## Historical perspective of the role of 5-HT in gut motility

It is well-established that the majority of 5-HT in the body is synthesized in the gut wall (Erspamer, 1954). Since this discovery, a number of studies have proposed that the release of serotonin from the GI tract may play an important role in the control of different neurogenic GI motility patterns, such as peristalsis, and migrating complexes in the small and large intestine (Büllbring and Lin, 1957; Bülbring and Lin, 1958; Bülbring et al., 1958; Grider et al., 1996; Jin et al., 1999; Heredia et al., 2009). Abundant *circumstantial* evidence has been presented to support this hypothesis. Evidence comes from the fact that (1) the largest quantity of serotonin in the body is synthesized in enterochromaffin cells in the mucosa (Erspamer, 1954), (2) high concentrations of 5-HT can be dynamically released from the mucosa (Bertrand, 2006; Keating and Spencer, 2010; Spencer et al., 2011) (3) exogenous 5-HT potently stimulates GI motility (Büllbring and Lin, 1957; Bülbring and Lin, 1958; Keating and Spencer, 2010; Spencer et al., 2011), and (4) numerous antagonists of 5-HT receptors can inhibit, or block peristalsis and reduce propulsion of contents (Grider et al., 1996; Kadowaki et al., 1996; Heredia et al., 2009), including rectal distension reflexes (Shimatani et al., 2003). Collectively, these studies provided a strong, albeit indirect case, that 5-HT may play an important role in GI motility.

**Figure 1:**
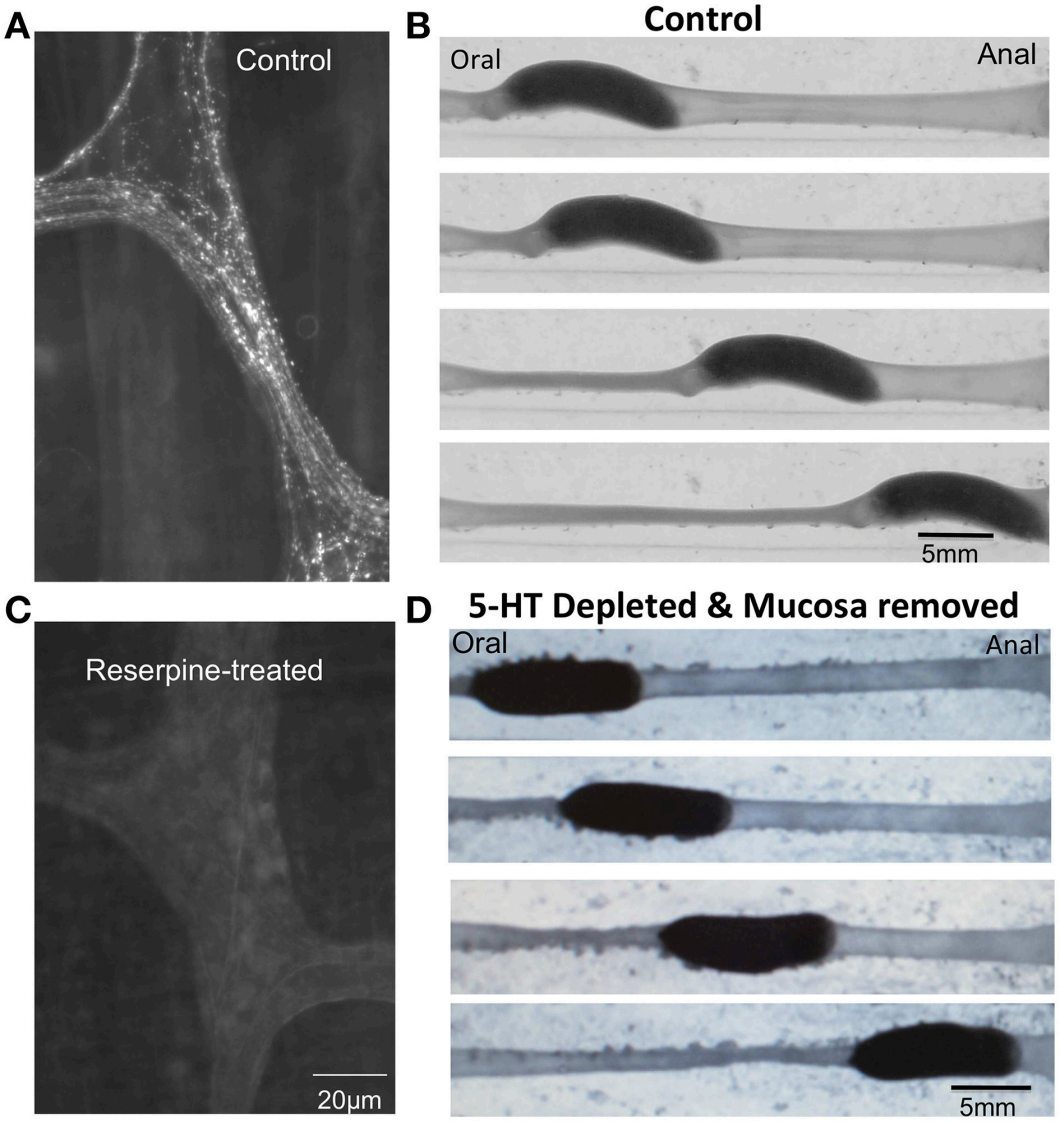
**(A)** Immunohistochemical staining of a myenteric ganglion with antibodies against 5-HT. Varicose 5-HT immunoreactive nerve axons ramify within myenteric ganglia and internodal strands.**(B)** Distension-evoked peristalsis elicited by insertion of an artificial fecal pellet. **(C)** After injection of reserpine into conscious guinea-pigs, all 5-HT is depleted from enteric ganglia. **(D)** Despite removal of all the mucosa and submucosal plexus and depletion of 5-HT from myenteric ganglia, distension-evoked peristalsis still reliably occurs.

**Figure 2:**
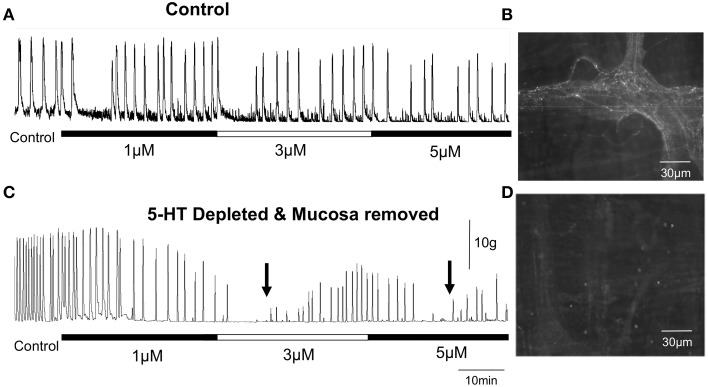
**Repetitive peristaltic contractions evoked by maintained colonic distension with a fixed artificial fecal pellet are only temporarily inhibited by 5-HT3 and 5-HT4 antagonists in isolated guinea-pig distal colon**. **(A)** Shows that increasing concentrations of ondansetron and SDZ-205-557 have only temporary effects on inhibiting distension-evoked peristaltic contractions in control distal colon. **(B)** Shows 5-HT is present in varicose nerve axons of myenteric ganglia. **(C)** In reserpine-treated guinea-pigs to deplete neuronal 5-HT and which also have their mucosa and submucosal plexus removed, maintained colonic distension by a fecal pellet continues to evoke repetitive peristaltic contractions. Simultaneous addition of increasing concentrations of ondansetron and SDZ-205-557 temporarily inhibited peristaltic contractions, which recovered in the continued presence of both antagonists. The two black arrows indicated when peristaltic contractions recover in the presence of both antagonists when applied at 3 μM and then 5 μM. **(D)** Reserpine treatment depleted all neuronal 5-HT from this specimen.

Despite seemingly convincing data supporting a role for endogenous 5-HT in the control of GI motility, the notion that endogenous 5-HT played a major role in control of GI motility was spectacularly revised in recent years, based on independent findings from different laboratories (Keating and Spencer, 2010; Yadav et al., 2010; Li et al., 2011; Spencer et al., 2011; Heredia et al., 2013; Sia et al., 2013a). These recent studies revealed that depletion of 5-HT from enteric neurons and complete removal of the mucosa and submucosal plexus did not prevent distension-evoked peristalsis (Figure 1) (Spencer et al., 2011; Sia et al., 2013a), nor CMMCs (Spencer et al., 2013). This is opposite to what one would expect if 5-HT was essential for these motor patterns. Then, Dr. Gershons' laboratory (Yadav et al., 2010; Li et al., 2011) revealed that deletion of the gene responsible for 5-HT synthesis in enterochromaffin (EC) cells (>95% of the 5-HT in the body) did not cause any decrease in transit *in vivo* (Yadav et al., 2010; Li et al., 2011). Finally, it was revealed that in segments of gut depleted of all detectable 5-HT, selective antagonists of 5-HT3 and 5-HT4 receptors still had the same, or greater, inhibitory effects on gut motility (Spencer et al., 2013) (see below, Figure 2).

## Previous misconceptions regarding the interpretation of the mechanisms of action of 5-HT3 antagonists on colonic motility

We made a conspicuous error (Bush et al., 2001) when interpreting the effects of a 5-HT3 antagonist (Alosetron) on propagating neurogenic motor patterns in the isolated mouse colon. We studied colonic migrating motor complexes (CMMCs) and found that they were potently abolished by the selective 5-HT3 receptor antagonist alosetron, at physiologically relevant concentrations (Bush et al., 2001). Based on this result, we speculated that 5-HT itself maybe important for the generation of CMMCs, by activating 5-HT3 receptors. This turned out to be an incorrect hypothesis because it is now known that CMMCs still occur when all 5-HT is depleted from the mouse colon (Spencer et al., 2013). Furthermore, 5-HT3 antagonists still had the same potent inhibitory effects on CMMCs in preparations of mouse colon that lacked all endogenous 5-HT (Spencer et al., 2013)—further refuting our original hypothesis (Bush et al., 2001). This revealed that the inhibitory effects of 5-HT3 antagonists on CMMCs occurred independently of endogenous 5-HT. In hindsight this result now makes sense, because no laboratory could ever find evidence that 5-HT is an enteric neurotransmitter in mouse colon (Furukawa et al., 1986; Nurgali et al., 2004) and of course, 5-HT is only synthesized in 1% of enteric neurons (Costa et al., 1996). The observation that CMMCs in mouse colon and peristaltic waves in guinea-pig colon (Spencer et al., 2011; Sia et al., 2013a) still occurred when all detectable 5-HT was depleted from the colon made it clear that 5-HT was not required for their generation and propagation, regardless of the effects of any antagonists. The major unanswered question then, was how do 5-HT3 antagonists block CMMCs in 5-HT depleted segments of colon? The most likely answer that has now emerged is that 5-HT3 and/or 5-HT4 receptors can be constitutively active and contribute to the background excitability of enteric neurons even in the absence of any 5-HT. And, when these receptors are blocked by 5-HT3 and 5-HT4 antagonists, they temporarily reduce the excitability of the neural network (Figure 2). Unequivocal evidence that 5-HT3 and 5-HT4 receptors are not required for distension-evoked peristalsis was demonstrated when in the continued presence of both antagonists, peristalsis recovers after a temporary blockade (Figure 2).

## Misconceptions regarding the functional role of neurochemicals synthesized in enteric neurons

Some investigators have assumed that the presence of a neurochemical in enteric neurons implies there must be a functional role of each of these neurochemical. This is an incorrect assumption. There are many (>20) different neurochemicals known to be synthesized in enteric neurons (including 5-HT, galanin, GABA, glutamate, CCK, etc.). But, most do not meet the criteria as “neurotransmitters” and lack any evidence they cause any post synaptic effector responses following neuronal stimulation. 5-HT is one such example. 5-HT is synthesized in < 1% of enteric neurons (Costa et al., 1996), but it does not cause postsynaptic potentials in enteric neurons of most species studied.

Another misconception that has been made is that 5-HT receptors can only be activated by their natural cognate ligand (5-HT). Evidence has been presented that in heterologously expressed receptor operated (Hu and Peoples, 2008) and G-protein coupled 5-HT receptors (Berthouze et al., 2005) can be activated without the presence of any exogenous 5-HT. This means that both ligand-gated and G-protein coupled 5-HT receptors can be constitutively active. Knowledge of this is mechanism is very important in potentially explaining the long-standing conundrum as to why 5-HT antagonists (like ondansetron and alosetron) can still potently inhibit GI motility in mice and other species, like rats and humans, where 5-HT is not a functional enteric neurotransmitter (Sia et al., 2013a). This knowledge would also now clearly explain why these 5-HT antagonists often have only temporary effects to block peristalsis, where peristalsis can recover in the continued presence of the antagonists (Sia et al., 2013b).

## Why 5-HT fails the criteria to be a neurotransmitter in the ENS of most species

According to the classic definition of a neurotransmitter, it is “*.a substance that is released at a synapse of by one neuron and that affects another cell, either neuron or effector organ, in a specific manner…”* (Kandel et al., 2000). Despite exhaustive attempts from different laboratories, intracellular electrophysiological recordings from enteric neurons in mice (Furukawa et al., 1986; Nurgali et al., 2004), rats (Brookes et al., 1988) and humans (Brookes et al., 1987) have not be able to record any fast synaptic potentials that could be attributed to endogenous 5-HT, even though clear evidence exists that 5-HT receptors are endowed on enteric neuronal cell membranes. In these latter three species, all fast synaptic potentials in enteric neurons are abolished, or almost blocked by nicotinic antagonists. We, and other laboratories, tried desperately to demonstrate reliable evidence that serotonin is a neurotransmitter in the enteric nervous system. But, we failed (Spencer and Smith, 2004). In our experiments, in Dr Smith's laboratory, all fast synaptic inputs were blocked by hexamethoniun and we found no evidence that 5-HT is a neurotransmitter in the colon of guinea-pigs (Spencer and Smith, 2004). In guinea-pig small intestine, small amplitude fast excitatory post synaptic potentials have been demonstrated to occur in about 10% of enteric neurons (Galligan et al., 2000; Nurgali et al., 2003; Furness, 2006). Perhaps the most interesting aspect of these findings is that despite 5-HT not being a neurotransmitter in human, rat, or mouse enteric nerves, 5-HT antagonists can clearly reduce intestinal transit in these species (Balfour et al., 2000; Bush et al., 2001). The mechanism how this works had remained elusive, until it was recently discovered that 5-HT3 and 5-HT4 receptors in the gut are likely to be constitutively active (Sia et al., 2013b). Another important possibility to explain the inhibitory effects of 5-HT3 antagonists on gut motility is that 5-HT3 antagonists block non-serotonergic receptors. In an interesting study on guinea-pig enteric neurons, fast synaptic potentials evoked by nerve stimulation and by ionophoretic application of exogenous acetylcholine could be reduced by 5-HT3 antagonists (Vanner and Surprenant, 1990). The IC-50 of the 5-HT3 antagonists ICS-930, GR 38032F, and MDL 7222 to inhibit 5-HT-mediated fast synaptic potentials was 12, 100 nM, and 3 μM, respectively. It was shown that these same 5-HT3 antagonists could inhibit acetylcholine-induced fast EPSPs in the same preparation although considerably greater concentrations (up to 100-fold higher IC-50 concentrations) in the micromolar range were required (Vanner and Surprenant, 1990). In our experiments, low nanomolar concentrations of the 5-HT3 antagonist ondansetron still potently inhibit CMMCs in isolated segments of whole mouse colon that are depleted of all 5-HT (Spencer et al., 2013).

## Why would a neurochemical (like 5-HT) be synthesized in the enteric nervous system if it does not function as a neurotransmitter?

There is now overwhelming evidence in mammals that a variety of proteins, receptors, neurotransmitters, ion channels, even whole organs have no essential function for survival on earth. For example, there is no essential function of nipples on men, the appendix in the GI tract, the coccyx bone, or tail that still develops in some humans, the helix in the ear, the tonsils, the arrector pili (goose bumps), wisdom teeth, plica semilunaris (nictitating membrane) in the eye lid, the sinus cavities in the skull, even the gall bladder is not essential. Humans can live successfully without any of these. The question that is then commonly raised is why are these cells, neurochemicals, receptors, even whole organs (e.g., gall bladder) synthesized in the body if they do not have an essential function for normal day-day life? The answer is that they are evolutionary vestiges. Evolutionary pressure and environmental change determine their relative importance in each species. It is entirely possible that different neurochemicals such as 5-HT, GABA, glutamate, CCK etc. which rarely, or never fulfill a functional role in the gut of healthy mammals, may become very important in disease states. However, it is important to recognize that a change in expression of “putative” enteric neurotransmitters, receptors, ion channels etc., during disease may be misinterpreted as being *causally-related* to the disease, rather than a *consequence* of the disease.

## Concluding remarks

There has been a paradigm shift in recent years in our understanding of endogenous 5-HT in GI motility and transit. The observation that endogenous 5-HT itself is not required for major GI motor patterns, including normal orderly transit of intestinal contents is a major change from original hypotheses; and a major step forward in gastroenterology (Yadav et al., 2010; Li et al., 2011). Another recent major breakthrough is that 5-HT antagonists retain their potent inhibitory effects on GI motility in segments of bowel depleted of all 5-HT (Sia et al., 2013b). This has led to that notion that 5-HT3 and 5-HT4 receptors in the GI tract are likely to exhibit constitutively active and that acute application of 5-HT3 and 5-HT4 antagonists can affect GI motility, despite the lack of all detectable 5-HT. We have now learnt from our mistakes and we take supreme caution when interpreting any effects of 5-HT antagonists on GI motility. Any future studies on the GI tract that demonstrate changes in cellular activity caused by antagonism of 5-HT receptors should entertain the possibility that the 5-HT receptors maybe constitutively active.

### Conflict of interest statement

The author declares that the research was conducted in the absence of any commercial or financial relationships that could be construed as a potential conflict of interest. The reviewer James J. Galligan and handling Editor Brian D. Gulbransen declared a current collaboration and the handling Editor states that the process nevertheless met the standards of a fair and objective review.

## References

[B1] Balfour J. A. Goa K. L. Perry C. M. (2000). Alosetron. Drugs 59, 511–518. discussion: 519–520. 10.2165/00003495-200059030-00008 10776833

[B2] Berthouze M. Ayoub M. Russo O. Rivail L. Sicsic S. Fischmeister R.. (2005). Constitutive dimerization of human serotonin 5-HT4 receptors in living cells. FEBS Lett. 579, 2973–2980. 10.1016/j.febslet.2005.04.040 15896782

[B3] Bertrand P. P. (2006). Real-time measurement of serotonin release and motility in guinea pig ileum. J. Physiol. 577, 689–704. 10.1113/jphysiol.2006.117804 16959854 PMC1890433

[B4] Brookes S. J. Ewart W. R. Wingate D. L. (1987). Intracellular recordings from myenteric neurones in the human colon. J. Physiol. 390, 305–318. 10.1113/jphysiol.1987.sp016702 2895177 PMC1192182

[B5] Brookes S. J. Ewart W. R. Wingate D. L. (1988). Intracellular recordings from cells in the myenteric plexus of the rat duodenum. Neuroscience 24, 297–307. 10.1016/0306-4522(88)90332-6 2452995

[B6] Büllbring E. Lin R. C. Y. (1957). The action of 5-hydroxytryptamine (5-HT) on peristalsis. J. Physiol. 138:12P.

[B7] Bülbring E. Lin R. C. Y. (1958). The effect of intraluminal application of 5-hydroxytryptamine and 5-hydroxytryptophan on peristalsis; the local production of 5-HT and its release in relation to intraluminal pressure and propulsive activity. J. Physiol. 140, 381–407. 13514713 PMC1358765

[B8] Bülbring E. Lin R. C. Y. Schofield G. (1958). An investigation of the peristaltic reflex in relation to anatomical observations. Q. J. Exp. Physiol. 43, 26–43. 10.1113/expphysiol.1958.sp001305 13494679

[B9] Bush T. G. Spencer N. J. Watters N. Sanders K. M. Smith T. K. (2001). Effects of alosetron on spontaneous migrating motor complexes in murine small and large bowel *in vitro*. Am. J. Physiol. Gastrointest. Liver Physiol. 281, G974–G983. 11557518 10.1152/ajpgi.2001.281.4.G974

[B10] Costa M. Brookes S. J. Steele P. A. Gibbins I. Burcher E. Kandiah C. J. (1996). Neurochemical classification of myenteric neurons in the guinea-pig ileum. Neuroscience 75, 949–967. 10.1016/0306-4522(96)00275-8 8951887

[B11] Erspamer V. (1954). Pharmacology of indole-alkylamines. Pharmacol. Rev. 6, 425–487. 13236482

[B12] Furness J. B. (2006). The Enteric Nervous System. Oxford: Blackwell Publishing.

[B13] Furukawa K. Taylor G. S. Bywater R. A. (1986). An intracellular study of myenteric neurons in the mouse colon. J. Neurophysiol. 55, 1395–1406. 3016211 10.1152/jn.1986.55.6.1395

[B14] Galligan J. J. Lepard K. J. Schneider D. A. Zhou X. (2000). Multiple mechanisms of fast excitatory synaptic transmission in the enteric nervous system. J. Auton. Nerv. Syst. 81, 97–103. 10.1016/S0165-1838(00)00130-2 10869707

[B15] Grider J. R. Kuemmerle J. F. Jin J. G. (1996). 5-HT released by mucosal stimuli initiates peristalsis by activating 5-HT4/5-HT1p receptors on sensory CGRP neurons. Am. J. Physiol. 270, G778–G782. 8967488 10.1152/ajpgi.1996.270.5.G778

[B16] Heredia D. J. Dickson E. J. Bayguinov P. O. Hennig G. W. Smith T. K. (2009). Localized release of serotonin (5-Hydroxytryptamine) by a fecal pellet regulates migrating motor complexes in murine colon. Gastroenterology 136, 1328–1338. 10.1053/j.gastro.2008.12.010 19138686 PMC2982771

[B17] Heredia D. J. Gershon M. D. Koh S. D. Corrigan R. D. Okamoto T. Smith T. K. (2013). Important role of mucosal serotonin in colonic propulsion and peristaltic reflexes: *in vitro* analyses in mice lacking tryptophan hydroxylase 1. J. Physiol. 591, 5939–5957. 10.1113/jphysiol.2013.256230 24127620 PMC3872763

[B18] Hu X. Q. Peoples R. W. (2008). The 5-HT3B subunit confers spontaneous channel opening and altered ligand properties of the 5-HT3 receptor. J. Biol. Chem. 283, 6826–6831. 10.1074/jbc.M707571200 18187416

[B19] Jin J. G. Foxx-Orenstein A. E. Grider J. R. (1999). Propulsion in guinea pig colon induced by 5-hydroxytryptamine (HT) via 5-HT4 and 5-HT3 receptors. J. Pharmacol. Exp. Ther. 288, 93–97. 9862758

[B20] Kadowaki M. Wade P. R. Gershon M. D. (1996). Participation of 5-HT3, 5-HT4, and nicotinic receptors in the peristaltic reflex of guinea pig distal colon. Am. J. Physiol. 271, G849–G857. 8944700 10.1152/ajpgi.1996.271.5.G849

[B21] Kandel E. Schwartz J. H. Jessel T. M. (2000). Principles of Neural Science. 4th Edn New York, NY: McGraw Hill.

[B22] Keating D. J. Spencer N. J. (2010). Release of 5-Hydroxytryptamine from the mucosa is not required for the generation or propagation of colonic migrating motor complexes Gastroenterology 138, 659–670. 10.1053/j.gastro.2009.09.020 19782081

[B23] Li Z. Chalazonitis A. Huang Y. Y. Mann J. J. Margolis K. G. Yang Q. M.. (2011). Essential roles of enteric neuronal serotonin in gastrointestinal motility and the development/survival of enteric dopaminergic neurons. J. Neurosci. 31, 8998–9009. 10.1523/JNEUROSCI.6684-10.2011 21677183 PMC4442094

[B24] Nurgali K. Furness J. B. Stebbing M. J. (2003). Analysis of purinergic and cholinergic fast synaptic transmission to identified myenteric neurons. Neuroscience 116, 335–347. 10.1016/S0306-4522(02)00749-2 12559090

[B25] Nurgali K. Stebbing M. J. Furness J. B. (2004). Correlation of electrophysiological and morphological characteristics of enteric neurons in the mouse colon. J. Comp. Neurol. 468, 112–124. 10.1002/cne.10948 14648694

[B26] Shimatani H. Kojima Y. Kadowaki M. Nakagawa T. Fujii H. Nakajima Y.. (2003). A 5-HT4 agonist mosapride enhances rectorectal and rectoanal reflexes in guinea pigs. Am. J. Physiol. Gastrointest. Liver Physiol. 285, G389–G395. 10.1152/ajpgi.00085.2003 12724131

[B27] Sia T. C. Flack N. Robinson L. Kyloh M. Nicholas S. J. Brookes S. J.. (2013a). Is serotonin in enteric nerves required for distension-evoked peristalsis and propulsion of content in guinea-pig distal colon? Neuroscience 240, 325–335. 10.1016/j.neuroscience.2013.02.061 23500097

[B28] Sia T. C. Whiting M. Kyloh M. Nicholas S. J. Oliver J. Brookes S. J.. (2013b). 5-HT3 and 5-HT4 antagonists inhibit peristaltic contractions in guinea-pig distal colon by mechanisms independent of endogenous 5-HT. Front. Neurosci. 7:136. 10.3389/fnins.2013.00136 23935564 PMC3732893

[B29] Spencer N. J. Nicholas S. J. Robinson L. Kyloh M. Flack N. Brookes S. J. (2011). Mechanisms underlying distension-evoked peristalsis in guinea-pig distal colon: is there a role for enterochromaffin (EC) cells? Am. J. Physiol. Gastrointest. Liver Physiol. 301, G519–G527. 10.1152/ajpgi.00101.2011 21700904

[B30] Spencer N. J. Nicholas S. J. Sia T. C. Staikopoulos V. Kyloh M. Beckett E. A. (2013). By what mechanism does ondansetron inhibit colonic migrating motor complexes: does it require endogenous serotonin in the gut wall? Neurogastroenterol. Motil. 25, 677–685. 10.1111/nmo.12136 23593931

[B31] Spencer N. J. Smith T. K. (2004). Mechanosensory S-neurons rather than AH-neurons appear to generate a rhythmic motor pattern in guinea-pig distal colon. J. Physiol. 558, 577–596. 10.1113/jphysiol.2004.063586 15146052 PMC1664963

[B32] Vanner S. Surprenant A. (1990). Effects of 5-HT3 receptor antagonists on 5-HT and nicotinic depolarizations in guinea-pig submucosal neurones. Br. J. Pharmacol. 99, 840–844. 10.1111/j.1476-5381.1990.tb13017.x 2141798 PMC1917554

[B33] Yadav V. K. Balaji S. Suresh P. S. Liu X. S. Lu X. Li Z.. (2010). Pharmacological inhibition of gut-derived serotonin synthesis is a potential bone anabolic treatment for osteoporosis. Nat. Med. 16, 308–312. 10.1038/nm.2098 20139991 PMC2836724

